# Selective inhibition of unfolded protein response induces apoptosis in pancreatic cancer cells

**DOI:** 10.18632/oncotarget.2051

**Published:** 2014-06-01

**Authors:** Wenwen Chien, Ling-Wen Ding, Qiao-Yang Sun, Lucia A Torres-Fernandez, Siew Zhuan Tan, Jinfen Xiao, Su Lin Lim, Manoj Garg, Kian Leong Lee, Shojiro Kitajima, Sumiko Takao, Wei Zhong Leong, Haibo Sun, Itay Tokatly, Lorenz Poellinger, Sigal Gery, Phillip H Koeffler

**Affiliations:** ^1^ Cancer Science Institute of Singapore, National University of Singapore, Singapore; ^2^ Cedars-Sinai Medical Center, UCLA School of Medicine, Los Angeles, California, USA; ^3^ Cancer Research Center, Edmond and Lily Safra Children's Hospital, Sheba Medical Center, Israel; ^4^ National University Cancer Institute, Singapore

**Keywords:** UPR, pancreatic cancer, IRE1α

## Abstract

Endoplasmic reticulum stress from unfolded proteins is associated with the proliferation of pancreatic tumor cells, making the many regulatory molecules of this pathway appealing targets for therapy. The objective of our study was to assess potential therapeutic efficacy of inhibitors of unfolded protein response (UPR) in pancreatic cancers focusing on IRE1α inhibitors. IRE1α-mediated XBP-1 mRNA splicing encodes a transcription factor that enhances transcription of chaperone proteins in order to reverse UPR. Proliferation assays using a panel of 14 pancreatic cancer cell lines showed a dose- and time-dependent growth inhibition by IRE1α-specific inhibitors (STF-083010, 2-Hydroxy-1-naphthaldehyde, 3-Ethoxy-5,6-dibromosalicylaldehyde, toyocamycin). Growth inhibition was also noted using a clonogenic growth assay in soft agar, as well as a xenograft *in vivo* model of pancreatic cancer. Cell cycle analysis showed that these IRE1α inhibitors caused growth arrest at either the G1 or G2/M phases (SU8686, MiaPaCa2) and induced apoptosis (Panc0327, Panc0403). Western blot analysis showed cleavage of caspase 3 and PARP, and prominent induction of the apoptotic molecule BIM. In addition, synergistic effects were found between either STF-083010, 2-Hydroxy-1-naphthaldehyde, 3-Ethoxy-5,6-dibromosalicylaldehyde, or toyocamycin and either gemcitabine or bortezomib. Our data suggest that use of an IRE1α inhibitor is a novel therapeutic approach for treatment of pancreatic cancers.

## INTRODUCTION

Pancreatic ductal adenocarcinoma is one of the most common causes of death from cancer in both males and females.[1] More than 80% of patients are diagnosed at an advanced stage; and the average 5-year survival rate is less than 5%[1]. One important feature of pancreatic cancer is the intense desmoplastic reaction around the tumors, which may impede delivery of therapeutic agents.[2] Gemcitabine and 5-fluorouracil are the standard treatment for advanced pancreatic cancers. [3, 4] and a large number of gemcitabine-based combinations have been/are being tested.[5, 6] However, to a large extent, chemotherapy is ineffective and novel therapeutic options are direly needed.[7]

Endoplasmic reticulum (ER) is an organelle where cellular processes occur such as lipid synthesis, calcium storage, and appropriate processing of membrane and secreted proteins for maturation. Stress such as hypoxia or oxidative stress can cause accumulation of misfolded proteins in ER lumen, which triggers unfolded protein response (UPR) for either ER homeostasis or apoptosis.[8] Adapting to ER stress, cells activate a dynamic UPR mechanism that has three major signaling pathways: IRE1α/XBP-1, ATF6, and PERK (pancreatic endoplasmic reticulum kinase). In response to ER stress, an initial cytoprotective mechanism is to release the ER chaperone GRP78 to facilitate protein folding. GRP78 is up-regulated in many cancers and is associated with poor survival.[9-11] However, when cells are unable to protect against ER stress, both intrinsic and extrinsic cell death pathways are activated, and severely damaged cells are removed.[12, 13] Additionally, after these sensor molecules (IRE1α/XBP-1, ATF6, and PERK) are released from GRP78, cascades of UPR signaling are activated to balance survival against damage caused by ER stress. Of note, the tumor microenvironment for pancreatic cancers is extremely rich in stroma and is hypoxic and deficient in metabolites.[14] This predisposes these tumors to ER stress and UPR activation. The dynamics of UPR in these rapidly growing hypoxic tumors present potential therapeutic option.[15-17]

Therapy based on targeting GRP78 and other UPR signaling has been shown to inhibit growth of tumors.[18, 19] Drugs activating ER stress have been use for clinical treatment of cancers. Bortezomib, a proteosome inhibitor, induces ER stress and has therapeutic efficacy in multiple myeloma.[20, 21] Bortezomib also induces ER stress in pancreatic cancer cells and suppresses the UPR in these cancer cells.[22] Bortezomib also synergizes with cisplatin causing apoptosis of pancreatic cancer cells which may be mediated through enhanced ER stress via increased expression of CHOP/GADD153 and BiP/GRP78.[23]

Another approach to induce pancreatic cancer cell death is to inhibit the repair of UPR. For example, an IRE1α inhibitor STF-083010 (STF) and its hydrolyzed product 2-Hydroxy-1-naphthaldehyde (HNA) block XBP-1 splicing, down-regulate XBP-1s expression and cause apoptosis in chronic lymphocytic leukemia (CLL) cells.[24] Furthermore, in multiple myeloma, inhibition of IRE1α-XBP-1s pathway by toyocamycin synergized with bortezomib to induce apoptosis.[25] In addition, high throughput screening identified salicylaldimine analogs as potent inhibitors of IRE1α endonuclease activity.[26] One salicylaldimine analog, 3-Ethoxy-5,6-dibromosalicylaldehyde (3ETH) inhibits XBP-1 splicing in myeloma cells both *in vitro* and in a murine model of ER stress *in vivo*.

As a result of these studies, we hypothesize that pancreatic cancers are under ER stress; and if we inhibit the protective mechanism of these cells against this stress by IRE1α inhibitors, these pancreatic cancer cells will undergo apoptosis suggesting a novel therapeutic approach to this rapidly fatal disease.

## RESULTS

### Anti-proliferative activity of IRE1α inhibitors in pancreatic cancer cell lines

**Figure 1:**
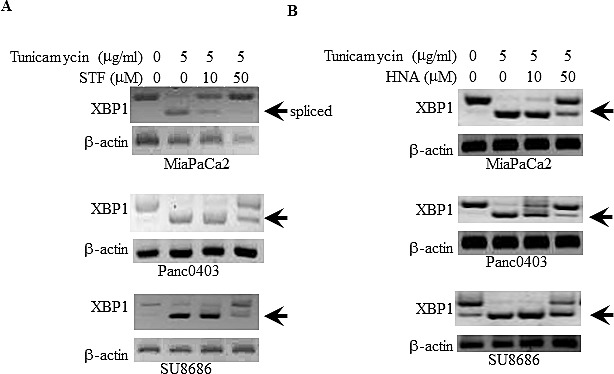
Suppression of tunicamycin-induced XBP-1 splicing by IRE1α inhibitors Three pancreatic cancer cell lines (MiaPaCa2, Panc0403, SU8686) were treated with either (A) STF or (B) HNA at either 10 or 50 μM for 6hr after pre-incubation with tunicamycin (5 μg/ml, 4hr). XBP-1 splicing was detected by PCR as described in the Materials and Methods. Beta-actin was examined as a loading control. Arrow demarks spliced form of XBP-1.

**Figure 2:**
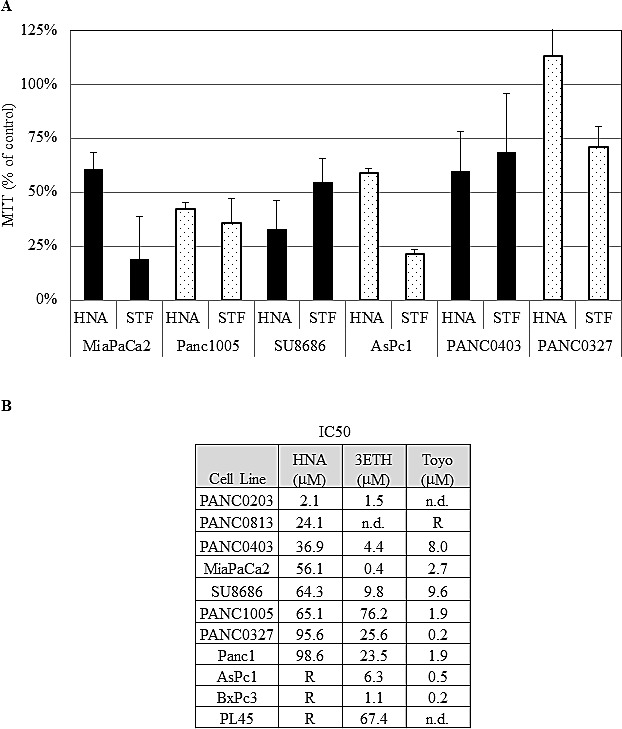
Anti-proliferative activities of IRE1αinhibitors (A) The effect of STF (50 μM) and HNA (50μM) on the cell growth of 6 pancreatic cancer cell lines compared to diluent treated controls (designated as 100%). Pancreatic cancer cell lines were treated with indicated drugs for 3 days, and cell viability was determined by MTT assays. (B) IC50s of HNA, 3ETH, and toyocamycin were determined using a series of concentrations (1 nM to 100 μM) of these drugs against a panel of pancreatic cancer cell lines using MTT assays. The IC50s were calculated with non-linear regression analysis using GraphPad Prism as described in Materials and Methods. R: Resistant at > 50 uM; n.d.: not done.

We initially examined the ability of two IRE1α inhibitors (STF and HNA) to inhibit the splicing of XBP-1 to XBP-1s in pancreatic cancer cell lines. Three pancreatic cancer cell lines (MiaPaCa2, Panc0403, SU8686) were pre-treated with tunicamycin to induce ER stress resulting in IRE1α activation and splicing of XBP-1 to XBP-1s. STF and HNA suppressed the levels of splicing in a dose-dependent manner (Fig.1). Since the inhibitory activity of STF in CLL was at the 50 μM range,[24] we expanded our analysis to 6 pancreatic cancer cell lines (MiaPaCa2, Panc1005, SU8686, AsPc1, Panc0403, Panc0327) using 50 μM of either STF or HNA. Other than Panc0327 cells, HNA was as potent as STF (Fig. 2A). The studies were then expanded and dose-responses of three different IRE1α inhibitors (HNA, 3ETH, toyocamycin) were tested against a panel of 11 pancreatic cancer cell lines (Panc0203, Panc0327, Panc0403, SU8686, MiaPaCa2, Panc1, Panc0813, AsPC1, BxPc3, Panc0203, Panc1005) using an *in vitro* proliferation assay (MTT). Most of the pancreatic cancer cell lines were sensitive to these 3 IRE1α inhibitors with a wide range of IC50s from 0.2 to 100 μM (Fig. 2B). Notably, three pancreatic cancer cell lines (AsPc1, BxPc3, PL45) were resistant to HNA even at 100 uM, but were sensitive to 3ETH and toyocamycin; while Panc0813 was sensitive to HNA, but resistant to toyocamycin (Fig. 2B). Together, these data suggested that these inhibitors had different modes of activity or metabolism within these cancer cells.

**Figure 3:**
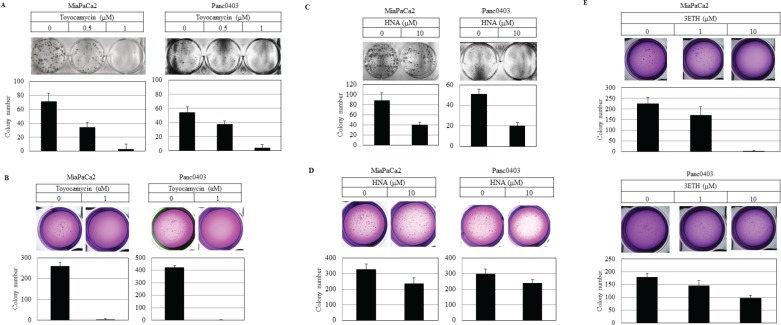
Colony formation of pancreatic cancer cells Pancreatic cancer cell lines (MiaPaCa2, Panc0403) were treated with drugs (toyocamycin [0.5, 1 μM], HNA [10 μM] or 3ETH [1, 10 μM]) for 14 days; and colony formation on plastic (A, C) and soft agar (B, D, E) was assessed. Representative plates are shown. Number of colonies on each plate was calculated with ImageJ; and numbers shown are average of duplicates from two independent experiments.

Colony formation on plastic and soft agar was examined using two pancreatic cancer cell lines (MiaPaCa2, Panc0403) after 14 days exposure to either HNA, 3ETH, or toyocamycin. Toyocamycin decreased clonal growth in a dose-dependent manner by both assays (Figs. 3A & 3B). Also, pancreatic cancer cells treated with HNA formed progressively fewer colonies on plastic (Fig. 3C), but to a lesser extent when assayed in soft agar (Fig. 3D). 3ETH was only examined in soft agar and also inhibited pancreatic clonal growth (Fig. 3E).

**Figure 4:**
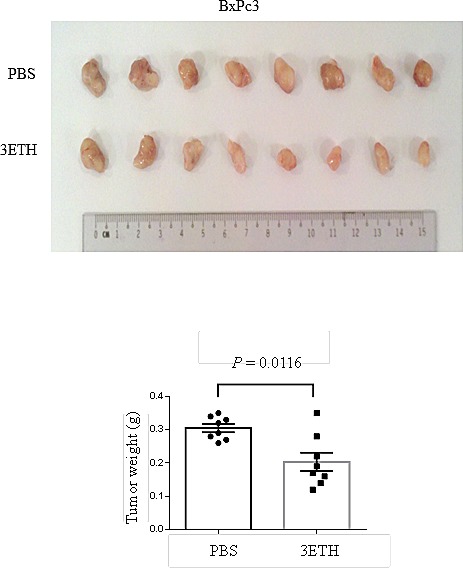
Effect of 3ETH on growth of BxPc3 human pancreatic cancer xenografts in NOD/SCID mice Two million BxPc3 cells were subcutaneously injected, and drug treatment was started at day 4. 3ETH was injected intraperitoneally three times a week for 4 weeks. Tumors were harvested and weights were measured. Top panel: tumors; Bottom panel: weights.

The anti-tumor activity of 3ETH on pancreatic cancer cell growth *in vivo* was examined using BxPc3 human tumors growing as xenografts in NOD/SCID mice. After tumors began their growth at day 4, mice were divided blindly into two groups and treated with either 20 mg/kg of 3ETH or vehicle (PBS) alone for 4 weeks. At conclusion of the study, tumors were carefully dissected and weighed. Mean weight of tumors was significantly greater in the control mice than the cohort treated with 3ETH (Fig. 4, *P*= 0.0016). We also tested HNA *in vi*vo by growing Panc0403 human xenograft in NOD/SCID mice. The difference in tumor weight between HNA-treated xenograft and control was less remarkable (*p* = 0.29, data not shown).

### Synergistic effects of IRE1α inhibitors combined with other therapeutic reagents, as well as the effect of hypoxia

**Figure 5:**
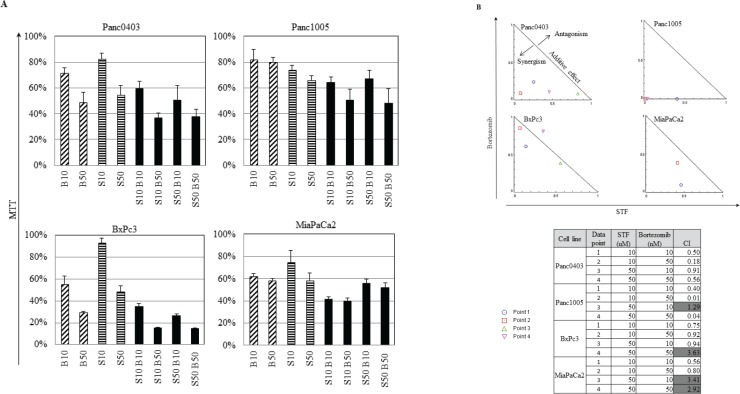
IRE1α inhibitor STF and proteasome inhibitor bortezomib synergistically inhibited the *in vitro* proliferation of pancreatic cancer cells (A) Four pancreatic cancer cell lines (Panc0403, Panc1005, BxPc3, MiaPaCa2) were cultured at different concentrations of drugs, and cell viability was measure by MTT assays. Drugs used were bortezomib (B10, B50: bortezomib 10, 50 nM) and STF (S10, S50: STF 10, 50 μM). (B) Isobologram analysis of combination of STF with Bortezomib from A (Panel B, top). CalcuSyn software was used to produce normalized isobolograms; and values below threshold line indicate synergistic combination; gray shaded boxes indicate non-synergistic combination (CI > 1) (Panel B, bottom).

Because the proteasome inhibitor bortezomib induces cell stress.[22] we investigated if the combination of IRE1α inhibitors with bortezomib has the capacity to act synergistically. Four pancreatic cancer cell lines (Panc0403, Panc1005, BxPc3, MiaPaCa2) were treated with different combination of bortezomib (10 or 50 nM) and STF (10 or 50 μM) (Fig. 5A). The normalized isobologram analysis demonstrated synergistic activity between 10 μM STF and either 10 or 50 nM bortezomib in all four cell lines (Fig. 5B, data points 1 and 2). Moreover, a higher concentration of STF (50 μM) attained synergy after addition of bortezomib either at a concentration of 10 nM when tested against BxPc3 cells (Fig. 5B, data point 3), at a concentration of 50 nM against Panc1005 cells (Fig. 5B, data point 4), and at either 10 or 50 nM against Panc0403 cells (Fig. 5B, data points 3 and 4).

**Figure 6:**
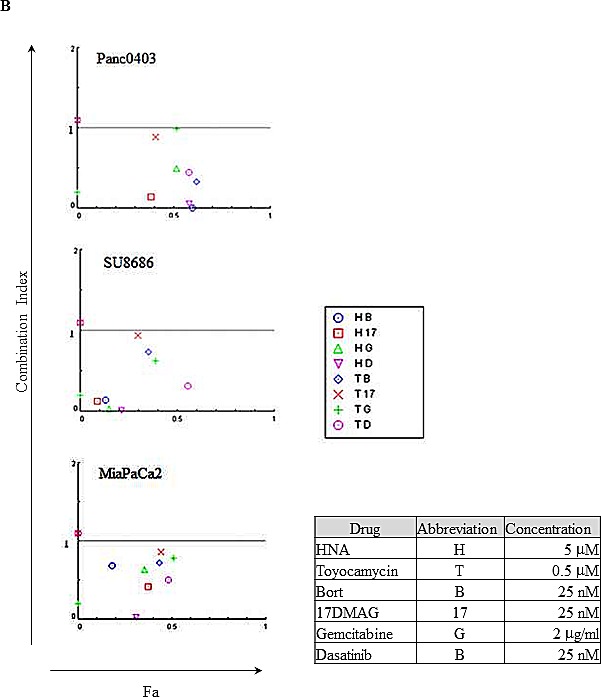
Combination index plot of HNA and toyocamycin with four different drugs (bortezomib, 17-DMAG, gemcitabine, dasatinib) Three pancreatic cancer cell lines (Panc0403, SU8686, MiaPaCa2) were incubated with drugs for 48h at concentrations indicated in Figure. Combination index (CI) plots were calculated with Calcusyn software as described in Materials and Methods. CI <1 indicates synergism between two drugs. Fa: the fraction that is affected or inhibited. B: bortezomib; 17: 17-DMAG; G: gemcitabine; D: dasatinib.

We further explored the combination of either HNA or toyocamycin with either bortezomib, 17-DMAG (heat shock protein inhibitor, 17-Dimethylaminoethylamino-17-demethoxygeldanamycin), gemcitabine (frequently used therapeutic agent), or dasatinib (src kinase inhibitor, FDA-approved for treatment of pancreatic cancer) in pancreatic cancer cells (Panc0403, SU8686, MiaPaCa2). Combination index (CI) was calculated and the CI plots showed synergistic activity of either HNA or toyocamycin combined with these drugs at concentrations shown (Fig. 6).

**Figure 7:**
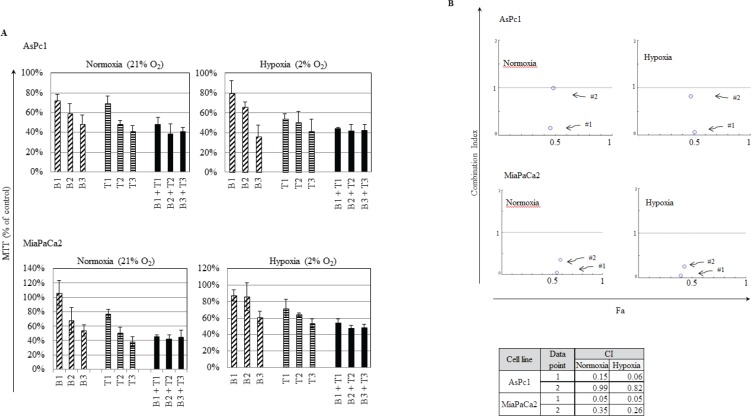
Synergistic effect when cultured in hypoxia Two pancreatic cancer cell lines (AsPc1, MiaPaCa2) were cultured in either normoxic (21% O_2_) or hypoxic (2% O_2_) conditions with different drug combinations for 48 hr. (A) Cell viability was measured by MTT assays. Drugs tested were bortezomib (B1: 1.6 nM; B2: 16 nM; B3: 166 nM) and toyocamycin (T1: 25 nM; T2: 250 nM; T3: 2500 nM). (B) Combination index (CI) was calculated as described in Materials and Methods. CI <1 indicates synergism between two drugs. Fa: affected fraction. Data point 1: 1.6 nM bortezomib, 25 nM toyocamycin); Data point 2: 16 nM bortezomib, 250 nM toyocamycin.

The fact that pancreatic tumors are usually densely embedded within stromal cells creating a hypoxic environment, we examined if the IRE1α inhibitor (toyocamycin) combined with bortezomib had the same efficacy at normoxic (21% O_2_) versus hypoxic (2% O_2_) conditions. Two cell lines (MiaPaCa2, AsPc1) were treated with the combination of toyocamycin (25, 250, or 2500 nM) and bortezomib (1.6, 16, or 166 nM) (Fig. 7A). CI plots showed that irrespective of the oxygen environment, synergistic growth inhibition prevailed against pancreatic cancer cells (Fig. 7B). The CI values were below 1 at the combination of 25 nM toyocamycin and 1.6 nM bortezomib (Fig. 7B, data point 1), or 250 nM toyocamycin and 16 nM bortezomib (Fig. 7B, data point 2).

### Mechanisms underlying the anti-proliferative effects of IRE1α inhibitors against pancreatic cancer cells:

**Figure 8:**
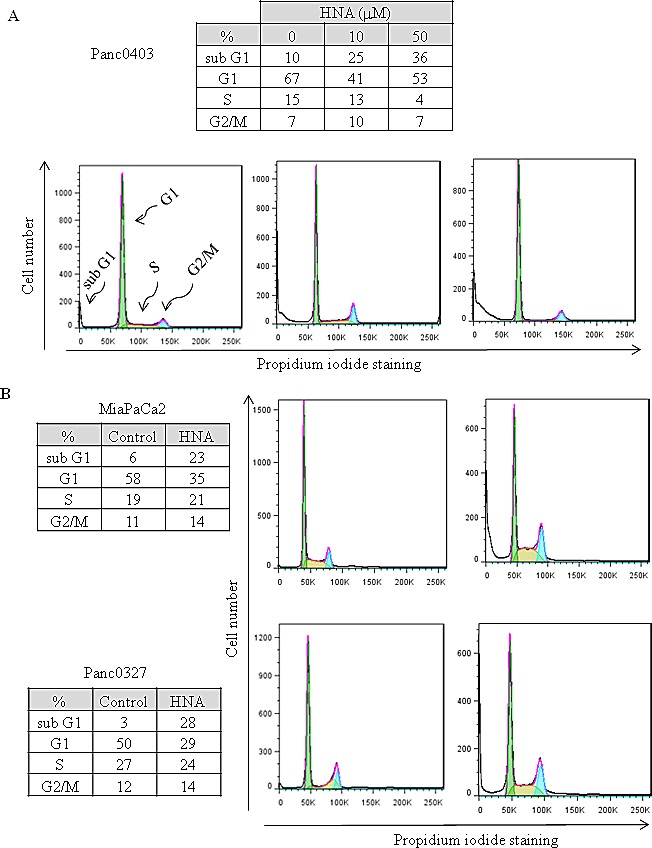
Cell cycle analysis Three pancreatic cancer cell lines (Panc0403, MiaPaCa2, Panc0327) were treated with HNA for 24 h; and DNA fractions were then analysed with flow cytometry. Cell cycle was fitted with Dean-Jett-Fox model in FlowJo as described in Materials and Methods. For each cell line, representative results from three independent experiments are shown.

Pancreatic cancer cells (Panc0403) cultured with HNA (10 and 50 μM, 24 hr) had a substantial pre-G1 fraction after exposure to 10 μM HNA (25%, apoptotic cells) and 50 μM HNA (36%, apoptotic cells) (Fig. 8A). Testing of two other pancreatic cancer cell lines (MiaPaCa2, Panc0327) treated with HNA (10 μM, 24 hr) also induced an increase of the pre-G1 fraction (23% and 28%, respectively, Fig 8B).

**Figure 9:**
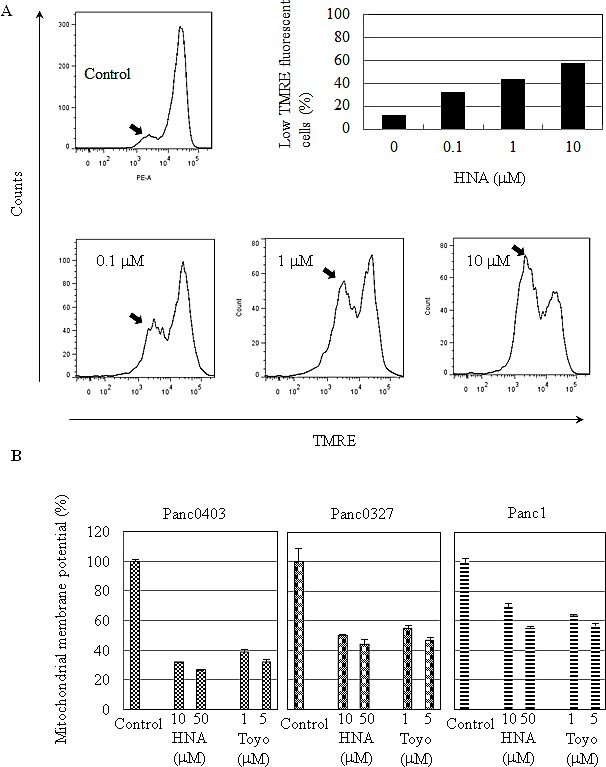
Mitochondrial membrane depolarization induced by HNA (A) Panc0403 pancreatic cancer cells were treated with HNA at 0.1, 1, 10 uM for 24 hr, and mitochondrial membrane potential was analysed by TMRE (tetramethyrhodamine ethyl ester percholarte) fluorescence. Histograms show the amount of TMRE sequestered by mitochondrial membrane. Low TMRE fluorescence (arrow) indicates decreased membrane potential. From the histogram, the portion of cells with low TMRE fluorescence (membrane potential) is summarized in the bar graph. (B) Three pancreatic cancer cell lines (Panc1, Panc0327, Panc0403) were treated with either HNA or toyocamycin at different concentrations for 24 hr and uptake of TMRE fluorescence by the cells was detected by a fluorescent plate reader. Each sample was run in duplicate, and the data represent the mean ± SD of two separate assays.

We wondered if these IRE1α inhibitors also effected mitochondria. The assay, TMRE, detects mitochondrial membrane depolarization. Panc0403 cells treated with increasing concentrations of either HNA (0.1 - 10 μM) or toyocamycin (1, 5 μM) showed decreasing mitochondrial membrane potential by either flow cytometer (Fig. 9A) or fluorescence microplate reader (Fig. 9B). Furthermore, two other pancreatic cancer cell lines (Panc1, Panc0327) treated with HNA (10, 50 μM) or toyocamycin (1, 5 μM) also displayed markedly decreased mitochondrial membrane potential (Fig. 9B).

**Figure 10:**
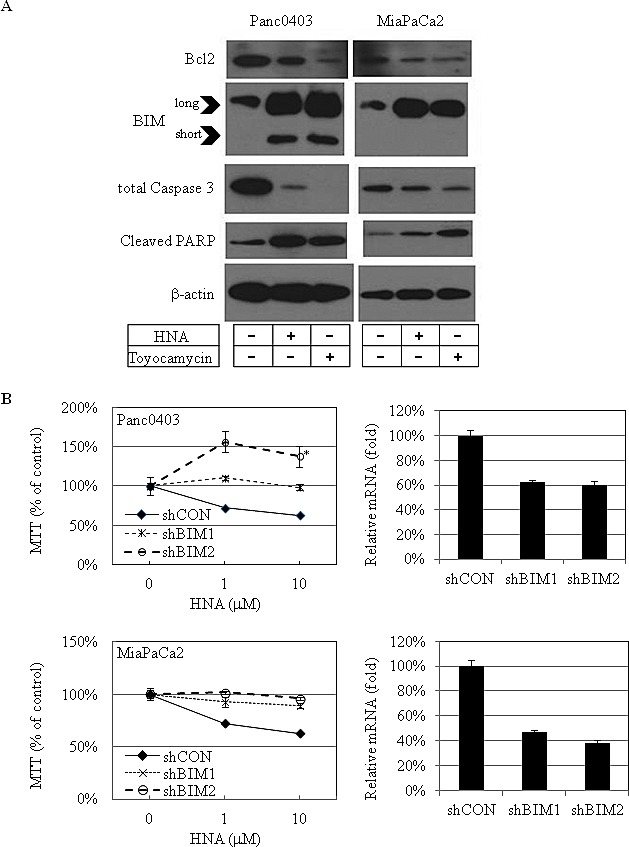
IRE1α inhibitor treatment: Apoptosis-related proteins in pancreatic cancer cells (A) Western blot analysis of apoptosis-related proteins in 2 pancreatic cancer cell lines (Panc0403, MiaPaCa2) after treatment with either HNA (10 μM) or toyocamycin (1 μM) for 24h. Beta-actin was loading control. Representative blots were from two independent experiments. (B) MTT assays of pancreatic cancer cell growth after knockdown of BIM. Two clones of pancreatic cancer cell lines stably infected with shRNA targeting BIM (shBIM1, shBIM2) were treated with HNA (1, 10 uM) for 48hr, and MTT assays was used to compared cell viability in experimental (shBIM1, shBIM2) versus scrambled shRNA control (shCON). *: *p* = 0.038 (left panel). Percent knock-down of BIM by shBIM1 and shBIM2 is shown on right panel.

Expression levels of proteins related to cellular apoptosis were examined in two pancreatic cancer cell lines (Panc0403, MiaPaCa2) after treatment with either HNA (10 μM) or toyocamcin (1 μM). Protein levels of the anti-apoptotic protein Bcl-2 decreased and the pro-apoptotic protein BIM increased (Fig. 10A). Both long and short forms of BIM were induced in Panc0403 cells, but only the long form was induced in MiaPaCa2 cells (Fig. 10A). Total caspase 3 protein levels decreased and cleaved PARP levels increased (Fig. 10A). Since BIM was markedly induced after HNA treatment, pancreatic cancer cells (MiaPaCa2, Panc0403) were infected with shRNA targeting BIM to examine the role of BIM in mediating the anti-proliferative activity of HNA. Two stable clones were selected from both cell lines infected with shBIM (shBIM1, shBIM2). Based on the real-time PCR, expression levels of BIM mRNA were similarly silenced in all stable pancreatic cancer cell lines containing shBIM (Fig. 10B), These experimental clones and control clone (one clone from scrambled shRNA - shCON) were treated with HNA (1, 10 uM), and cell viability were determined by MTT assays. Knockdown of BIM caused the pancreatic cancer cells to become more resistant to killing by HNA with Panc0403 with stable shBIM1 having the greatest resistance to HNA ((*p* = 0.038, Fig. 10B). This suggests pancreatic cancer cell death by IRE1α inhibitors is partially attributed to induction of BIM.

Since BIM can be transcriptionally activated by CHOP in UPR,[27] we examined CHOP mRNA levels after treatment with IRE1α inhibitors in four pancreatic cancer cell lines (MiaPaCa2, Panc0403, SU8686, AsPc1). As expected, pre-treatment with tunicamycin induced CHOP expression which was further increased by the addition of either STF or HNA (Fig. 10A).

DNAJB9 is one of the UPR target genes which transcriptionally activated by XBP-1s.[28] In pancreatic cancer cells treated with either STF or HNA, levels of DNAJB9 mRNA were suppressed after initial induction by tunicamycin (Fig. 10B). We also examined the transcription factor ATF4 of the PERK branch of UPR, and found that expression levels of ATF4 were not affected in two cell lines (MiaPaCa2, Panc0403), but were induced substantially in two other (SU8686, AsPc1) after either STF or HNA treatment (Fig. 10C). This suggests differential cellular response to these compounds.

**Figure 11:**
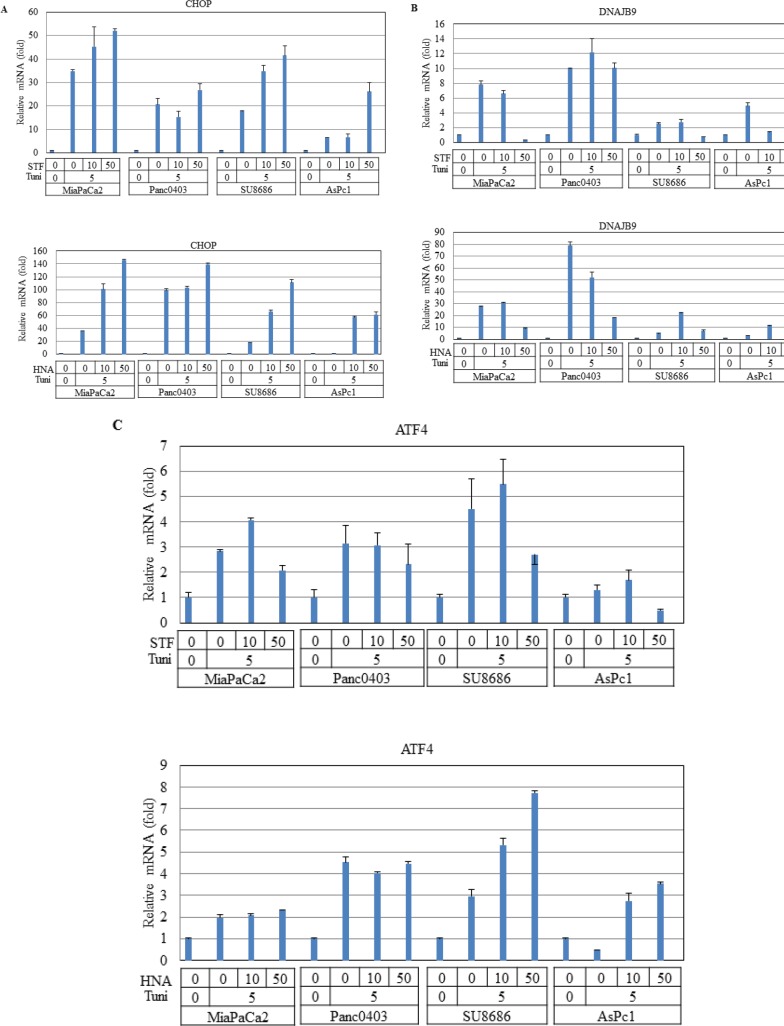
Effect of STF or HNA on expression of UPR target genes in pancreatic cancer cells Messenger RNA expression levels of (A) CHOP (B) DNAJB9 (C) ATF4 were examined by real-time PCR in four pancreatic cancer cells (MiaPaCa2, Panc0403, SU8686, AsPc1) after treatment with either STF or HNA (10 or 50 μM, 6hr). Tuni: tunicamycin (5 μg, 4 h) was added as a pre-treatment. Results are means ± SD of two independent experiments done in triplicates.

We also examined levels of several other key proteins associated with cellular ER stress and cell growth. Phosphorylated Erk and its upstream regulator PDK1, were down-regulated and phosphorylation of JNK was increased after HNA treatment in two of the pancreatic cancer cell lines (Fig. 11A). A reactive oxygen species (ROS) sensor thioredoxin binding protein (TXNIP) and thioredoxin (TXN) were evaluated in pancreatic cancer cells (Panc0403, MiaPaCa2) following treatment with either HNA or 3ETH. Messenger RNA expression levels of TXNIP were up-regulated while TXN levels were down-regulated by HNA and 3ETH (Fig. 11B).

## DISCUSSION

The ER plays an important role in the secretory pathway in which proteins undergo post-translational modification. Overload of nascent proteins or mis- and un-folded proteins induces ER stress and activates UPR. Studies have found that a histone deacetylase inhibitor such as panobinostat induces apoptosis in hepatocellular carcinoma associated with up-regulation of ER stress markers (Grp78, eIf2a, and XBP-1).[29] Treatment of chronic lymphocytic leukemia and multiple myeloma cells with brefeldin A, an inhibitor of ER to Golgi protein transport cause a decrease in VEGF secretion and abnormal ER swelling; and subsequently activation of caspases, and cell death.[30] UPR is an intricate process in the face of cellular ER stress. Initial UPR attempts to restore ER homeostasis is by temporarily suspending protein translation. In addition, ER stress stimulates IRE1α to splice XBP-1 mRNA resulting XBP-1s.[31] XBP-1s becomes an active transcription factor enhancing synthesis of genes involved in protecting the cells from changing levels of ER stress and unfolded poteins.

Pancreatic cancer remains a catastrophic disease with a 5-year survival rate of 5%. First-line therapy with either gemcitabine or gemcitabine-based chemotherapeutic combinations provides small clinical improvements, but new effective treatment options are clearly needed. We examined the effect of four IRE1α inhibitors (STF, HNA, 3ETH, toyocamycin) against a panel of 11 pancreatic cancer cell lines using a MTT liquid culture assay and found a wide range of drug sensitivities, ranging from 2 to 100 μM. We then employed a much more sensitive assay, clonogenic growth in soft agar which showed that these pancreatic cancer cells were very sensitive to growth inhibition by IRE1α inhibitors. Importantly, the IRE1α inhibitor (3ETH) also decreased the proliferation of human pancreatic cancer xenografts growing *in vivo* (70% growth compared to control cells, *p* = 0.0116). The data suggest IRE1α inhibitors significantly reduce growth of pancreatic tumors.

In further experiments, synergism was found by combining IRE1α inhibitors with FDA-approved agents such as either bortezomib, 17-DMAG, gemcitabine, or dasatinib. Our findings indicate that selective inhibition of ER stress by IRE1α inhibitors could curb pancreatic cancer cell growth in drug combination. Pancreatic cancers are extremely rich in stromal cells and are hypovascular, suggesting that the cancer cells are hypoxic. In view of this landscape, we explored the drug combination of bortezomib and toyocamycin in hypoxic conditions. Synergism was sustained when tested in hypoxic condition (2% O_2_).

Our mechanistic studies of how IRE1α inhibitors decrease pancreatic cancer cell proliferation found these agents robustly induced prominent levels of BIM. In contrast, silencing of BIM rendered pancreatic cancer cells less sensitive to killing by IRE1α inhibitors. Attenuation of cell death by BIM knockdown was partial and in some clones not statistically significant. This may be due to incomplete silencing of BIM as well as involvement of additional pathways important in cell death mediated by IRE1α inhibitors. We found the extra-long form of BIM (BIM-EL) was induced in two pancreatic cancer cell lines (Panc0403 and MiaPaCa2). The pro-apoptotic BIM-EL can be phosphorylated and subsequently degraded in the proteasome when ERK signaling is activated.[32, 33] In addition, phosphorylation of BIM by ERK reduces its pro-apoptotic activity by preventing binding of BIM to BAX.[34] On the other hand, phosphorylation of BIM by JNK at a different site activates the apoptotic activity of BIM and induces BAX-dependent apoptosis[35]. Our treatment of pancreatic cancer cells with IRE1α inhibitors decreased levels of phosphorylated ERK and increased levels of phosphorylated JNK. These data suggest upon treatment with IRE1α inhibitors, BIM was activated by JNK phosphorylation as well as heterodimerization with BAX to promote cell apoptosis of pancreatic cancer cells.

CHOP is another primary target of induction of cell death after stress activation of ER.[27] It can be activated transcriptionally through all three branches of UPR: IRE1α, PERK/ATF4, and ATF6.[36] Treatment of pancreatic cancer cells with IRE1α inhibitors still caused induction of CHOP, suggesting CHOP was activated through ATF4 and ATF6. Our real-time PCR results suggest the ATF6 pathway was stimulated. Also, a previous study showed that through DR5 activation, the JNK pathway induced CHOP and the cell death pathway.[37] As mentioned above, we showed that phosphorylation of JNK was up-regulated after exposure of the pancreatic cancer cells to an IRE1α inhibitor. This suggests that pancreatic cancer cell death by IRE1α inhibitors may also involve phosphorylation levels of JNK, which induces CHOP.

Examination of mitochondrial membrane potential revealed pancreatic cancer cell death caused by IRE1α inhibitors may also involve the intrinsic mitochondria-mediated apoptosis pathway. Mitochondrial membrane potential was lost when pancreatic cancer cells were treated with IRE1α inhibitors. Loss of mitochondrial membrane potential can lead to swelling of mitochondrial membrane and release of cytochrome C to cytosol, which activates caspase 3 and apoptosis.[38] Congruent with activation of this pathway, cleavage of caspase 3 and cell death occurred after exposure of the pancreatic cancer cells to IRE1α inhibitors.

ROS scavenger TXN and its interacting protein TXNIP play an important role in regulating oxidative stress[39] and over-expression of TXNIP will induce G0/G1 cell cycle arrest.[40] Crosstalk between ER stress and ROS has previously been suggested.[41, 42] Our data showed that TXNIP was induced and TXN was reduced after pancreatic cancer cells were treated with IRE1α inhibitors. These results suggest that IRE1α inhibitors may induce ROS helping to mediate cell death.

**Figure 12:**
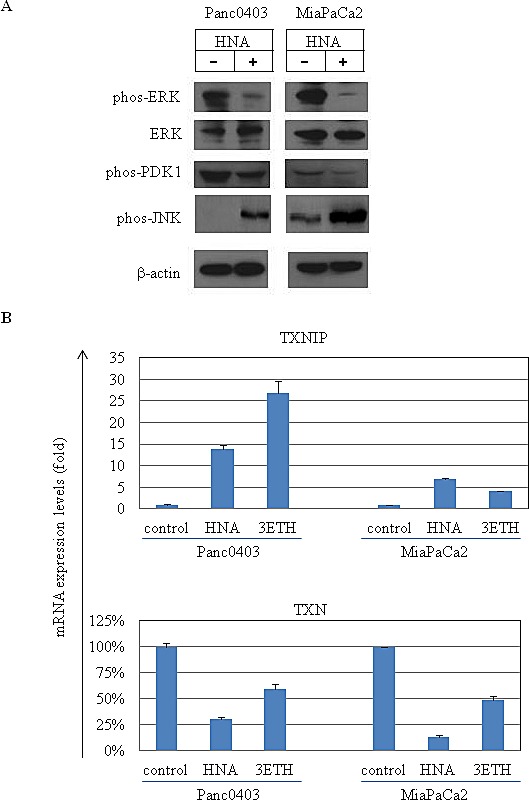
Protein and RNA expression profiles of pancreatic cancer cell lines after treatment with HNA and 3ETH (A) Western blot analysis of 2 pancreatic cancer cell lines treated with HNA (10 μM, 24hr). Antibodies used were phos-Erk (phosphorylated Erk), total Erk, phos-PDK and phos-pJNK. Beta-actin served as loading control. (B) Expression levels of TXNIP (thioredoxin binding protein) and TXN (thioredoxin) mRNA in Panc0403 and MiaCaPa2 cells after HNA and 3ETH treatment (1 μM, 24hr) by real-time PCR. Results are mean ± SD of two independent experiments done in triplicates.

Based on our findings, we proposed a scheme to elucidate the anti-proliferative activities of IRE1α inhibitors (STF, HNA, 3ETH, toyocamycin) in pancreatic cancer cells. IRE1α inhibitors caused overwhelming ER stress, which induced CHOP expression and subsequently BIM activation (Fig. 12, step 1). Perturbation of ER stress may regulate ROS through up-regulation of TXNIP and subsequently help trigger apoptosis by modulation of mitochondrial structure and functions (Fig. 12, step 2). On the other hand, TXNIP can directly activate phosphorylation of JNK and promote apoptosis (Fig. 12, step 3). Multiple events induced by IRE1α inhibitors including BIM activation, mitochondrial membrane depolarization, and caspase 3 activation lead to pancreatic cancer cell death. Our findings indicate that selective inhibition of ER stress by IRE1α inhibitors may have a therapeutic role in the management of pancreatic cancer.

## METHODS

### Cell lines and cell culture

Pancreatic cancer cell lines (Panc1, Panc0203, Panc0327, Panc0403, Panc0813, Panc1005, AsPc1, BxPc3, MiaPaCa2, PL45, SU8686) were obtained from ATCC (Manassas, VA). They were cultured in RPMI-1640 supplemented with 10% fetal bovine serum and maintained at 5% CO_2_ and 37°C. For induction of hypoxia, cells were incubated in temperature-controlled hypoxic culture chamber at 1% O_2_, 5% CO_2_, and 94% N_2_.

### MTT assays

Three thousand cells were seeded in 96-well plates overnight and drug treatment started the next day. After an incubation period, MTT (3-(4,5-dimethylthiazol-2-yl)-2,5-diphenyltetrazolium bromide) was added to cells and cultured at 37°C for 4 hr followed by stop solution (4 mM HCl, 0.1% Nondet P40 in isopropanol) which was added to dissolve MTT. The plates were read with a spectrophotometer at 590 nm absorbance with reference at 630 nm. IC50 values were calculated using GraphPad Prism (La Jolla, CA).

### Colony formation assays

For colony formation on plastic, 800 cells were seeded in 6-well plates overnight, and drugs were applied to the cultured cells on the second day. After 14 days, culture medium was removed, and cells were briefly rinsed with PBS. Colonies were stained with crystal violet (0.2%). For clonogenic growth in soft agar, 3,000 or 5,000 cells/well in 6-well plates were cultured in 0.35% low melting agarose either with or without drugs on top of a bottom layer of 0.5% agarose. Cells were cultured for either 14 days or when the colonies were large enough for enumeration. Colonies were stained with 1:50 Gentin Violet for 20 min and rinsed with PBS until the colonies were easily detected. Colonies were photographed and counted with ImageJ (http://rsbweb.nih.gov).

**Figure 13:**
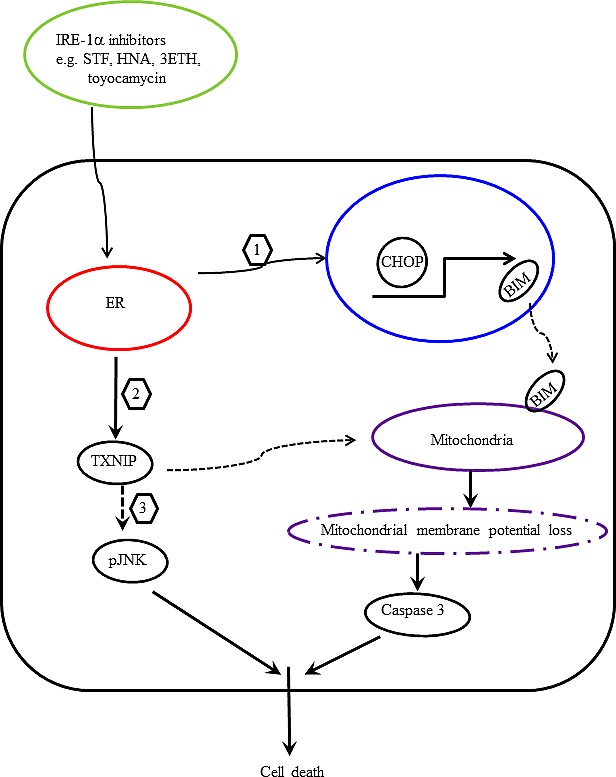
Proposed scheme of IRE1 α-induced pancreatic cancer cell death Treatment of pancreatic cancer cells with IRE1α inhibitors enhances ER stress and (1) activates CHOP transcription, which in turn transactivates BIM. Translocation of BIM to mitochondria enhances apopotosis. (2) Modulation of ROS by ER stress up-regulates TXNIP, which causes loss of mitochondrial membrane potential and activates caspase 3 and apoptosis. (3) In addition, TXNIP activates JNK phosphorylation and promotes apoptosis.

### Animal studies

BxPc3 pancreatic cancer cells (2x10^6^ cells/100 μl) mixed with matrigel at 1:1 ratio were injected subcutaneously into both flanks of NOD/SCID mice. Drug treatment was started four days later. Drug (3ETH) or control diluent (PBS) was injected intraperitoneally three times a week for a total of four weeks. Weight and size were measured after harvesting the tumors.

### Studies of drug combination

Results from MTT assays of different combinations of drugs were analyzed by Calcusyn (Biosoft, UK) for cumulative effects as expressed by isobolograms. An isobologram is a graph indicating the equipotent combinations of different doses of two drugs. Isobologram can clearly show additive, synergistic, or antagonistic effects at different dose levels. A combination index (CI) plot is a Fa-CI plot in which CI <1, =1, >1 indicate synergism, additive effect, and antagonism, respectively. Fa: the fraction that is inhibited by the drug.[43]

### Cell cycle analysis

After drug treatment, cells (1x10^6^/ml) were fixed with 70% ethanol at −20°C for 30 min. Cells were washed with PBS three times and stained with 40 ug/ml propidium iodide containing 500 ng/ml RNase A. Ten thousand events per sample were acquired on LCRII (BD Biosciences, Franklin Lakes, NJ) and analyzed with FlowJo software (Ashland, OR).

### Mitochodrial membrane potential analysis

Binding and accumulation of TMRE (tetramethyrhodamine ethyl ester percholarte) in mitochondria is driven by mitochondrial membrane potential. Depolarization of mitochondria membrane potential leads to loss of TMRE accumulation and decrease in TMRE staining. Mitochondrial membrane potential assessment kit (SeroTec, Oxford, UK) was used following manufacturer's instruction. Membrane potential was measured at 488 nm excitation and 670 nm emission with either flow cytometer or fluorescent plate reader. Pancreatic cancer cells [Panc1 (1x10^6^)] seeded in 10-cm dishes overnight, and then were treated with HNA (0, 1, 10 μM) for 24 hr. After drug treatment, cells were trypsinized, re-suspended in TMRE working solution to a density of 1x10^6^ cells/ml, and incubated for 20 minutes in a 37°C CO_2_ incubator. After incubation, cells were analyzed with flow cytometry. Histograms measured the proportion of mitochondria that were depolarized as indicated by a decrease in fluorescence. Three pancreatic cancer cell lines (Panc1, Panc0327, Panc0403) plated in 96-well plates were treated with drugs for 24hr, and mitochondrial membrane potential was measured with a fluorescent plate reader. Loss of membrane potential was detected by comparing the fluorescence against the average 575 nm fluorescence signal in cells.

### Western blot analysis

Following drug treatment, cells were directly lyzed with lysis buffer (Thermo Fisher Scientific, Waltham, MA) containing a protease inhibitor cocktail (Millipore, Billerica, MA) and scraped off of the culture dishes. After 10 min incubation on ice, the lysates were centrifuged at 10,000xg for 10 min at 4°C. Total protein concentration from the supernatants was determined by BCA assay (BioRad Laboratories, Hercules, CA). Thirty micrograms of protein were resolved on SDS-PAGE followed by transfer to PVDF (Millipore). Membranes were blocked with 5% non-fat milk and incubated with antibodies. Antibodies were from Santa Cruz (Santa Cruz, CA), Cell Signaling (Boston, MA), Sigma Aldrich (St. Louis, MO). ECL reagents (GE Healthcare Life Sciences, Uppsala, Sweden) were used to detect the proteins.
